# Combining Biomarkers for the Diagnosis of Metastatic Melanoma

**DOI:** 10.3390/jcm13010174

**Published:** 2023-12-28

**Authors:** Tünde Várvölgyi, Eszter Anna Janka, István Szász, Viktória Koroknai, Tünde Toka-Farkas, Imre Lőrinc Szabó, Beatrix Ványai, Andrea Szegedi, Gabriella Emri, Margit Balázs

**Affiliations:** 1 Department of Dermatology, MTA Centre of Excellence, Faculty of Medicine, University of Debrecen, 4032 Debrecen, Hungary; varvolgyi.tunde@med.unideb.hu (T.V.); janka.eszter@med.unideb.hu (E.A.J.); toka-farkas.tunde@med.unideb.hu (T.T.-F.); szabo.imre.lorinc@med.unideb.hu (I.L.S.); vanyai.beatrix@med.unideb.hu (B.V.); aszegedi@med.unideb.hu (A.S.); gemri@med.unideb.hu (G.E.); 2 HUN-REN-UD Allergology Research Group, University of Debrecen, 4032 Debrecen, Hungary; 3 HUN-REN-UD Public Health Research Group, University of Debrecen, 4028 Debrecen, Hungary; szasz.istvan@med.unideb.hu (I.S.); koroknai.viktoria@med.unideb.hu (V.K.); 4 Department of Public Health and Epidemiology, Faculty of Medicine, University of Debrecen, 4028 Debrecen, Hungary

**Keywords:** metastatic melanoma, osteopontin, S100B

## Abstract

The early detection of melanoma relapse can improve patient survival; thus, there is a great need for easily accessible biomarkers that facilitate the diagnosis of metastatic disease. We investigated the diagnostic effect of blood biomarkers such as lactate dehydrogenase (LDH), S100B, and osteopontin in the detection of metastases. Clinical data and peripheral blood samples of 206 melanoma patients were collected (no metastasis, N = 120; metastasis, N = 86). The discriminative power of blood biomarkers, patient demographics, and clinicopathological parameters of primary melanomas were evaluated using univariate and multivariate logistic regression models and receiver operating characteristic (ROC) analysis to determine the area under the curve (AUC). Plasma osteopontin levels showed a significant and independent effect on the probability of metastasis, similar to serum S100B levels. In addition, the location of the primary tumor on the lower extremities and the American Joint Committee on Cancer (AJCC) categories pT2b-3a, pT3b-4a, and pT4b were associated with the diagnosis of metastasis. Importantly, the combination of the three blood biomarkers and primary tumor localization and AJCC pT category yielded excellent discrimination (AUC: training set: 0.803; validation set: 0.822). In conclusion, plasma osteopontin can be classified as a melanoma biomarker; moreover, by combining clinicopathological prognostic variables, the diagnostic effect of blood biomarkers in the detection of metastatic melanoma can be improved.

## 1. Introduction

Melanoma is a malignant tumor of the melanocytes, predominantly of the skin, accounting for about 3% of malignant tumors. Its prevalence in the Caucasian race has increased significantly worldwide over the last 4 decades [1]. The number of diagnosed cases is increasing by 4–8% per year [2]. Although it accounts for less than 5% of all malignant skin tumors, nearly 73% of skin cancer-related deaths are due to melanoma [3,4]. Melanoma malignum is known to have a high risk of lymph node and haematogenous metastasis. Early metastases are those that typically develop within 3 years after diagnosis, but late metastases can also occur, even after 10 or more years of asymptomatic disease [5]. Primary tumor thickness (using the Breslow score) and the presence or absence of ulceration are the two main histological prognostic parameters [6]. While awareness campaigns and advancements in therapy have led to improvements in melanoma survival [7], there still exist unmet medical needs. There is an increasing need for easily available biomarkers from peripheral blood to facilitate the early detection of relapse and to improve overall survival. Serum or plasma biomarkers serve a vital role in treatment planning for metastatic disease, prognostic prediction, and monitoring therapy efficacy.

One established biomarker is serum lactate dehydrogenase (LDH), which is incorporated into the TNM (tumor/node/metastasis) staging of melanoma [5]. LDH levels exhibit a strong correlation with the tumor size and the extent of necrosis. However, it lacks specificity for melanoma, as elevated LDH levels can also occur in other malignancies, as well as in conditions such as myocardial infarction, haemolysis, and hepatitis [8,9]. LDH has consistently been demonstrated as the most robust prognostic factor in stage IV melanoma. However, its sensitivity and specificity could not achieve that of the S100B protein. S100B has garnered increased attention among researchers as a diagnostic marker for melanoma, due to its significantly higher expression and secretion in malignant tissue compared to healthy tissue. Notably, the elevated serum level of S100B has a higher specificity for disease progression compared to LDH [10].

According to literature data, osteopontin (OPN) also appears to be a promising marker for metastatic melanoma. In our previous study, we showed that OPN expression was increased in both the mRNA and protein levels in thick melanomas and was associated with ulceration [11]. In addition, we recently reported a significant increase in OPN (mRNA) expression in cell lines developed from melanoma metastases when compared to cell lines developed from primary melanomas [12]. In a clinical study, a significantly elevated OPN plasma concentration was found in patients with metastatic melanoma compared to non-metastatic patients [13].

The aim of the present study was to investigate the diagnostic effectiveness of three markers—LDH, S100B, and OPN—in our patient population, in comparison with the existing literature data regarding the identification of metastatic disease

## 2. Materials and Methods

### 2.1. Study Population

A total of 206 melanoma patients between 4 April 2019 and 22 September 2022 were included in the retrospective study. The data for this study were obtained from the University of Debrecen’s clinical database (MedSolution and UDMED). The study was approved by the Medical Research Council Ethic Committee (certificate number: IV/1711-4/2021/EKU). Patient data, including age and sex, histological subtype of primary melanoma, the Breslow tumor thickness, ulceration status, localization, Clark invasion level, primary tumor (T) stage (pT stage) according to the 8th edition American Joint Committee on Cancer (AJCC) TNM classification for melanoma [5], as well as serum S100B and LDH levels were recorded. Peripheral blood samples were collected from 206 melanoma patients (no metastasis, N = 120; metastasis, N = 86). The serum concentrations of S100B (chemiluminescent immunoassay LIAISON^®^ S100, Madison, WI, USA) and LDH (automated colorimetric assay) were routinely measured during the follow-up of patients every three months. Patients in the metastatic group were diagnosed with PET-CT, three-region CT, or soft tissue ultrasound within three months prior to the blood sampling date. The metastatic melanoma group included (1) patients not receiving any metastatic melanoma drugs yet but with confirmed metastasis and (2) metastatic patients with BRAF plus MEK inhibitor or immune checkpoint inhibitor therapy. The metastasis-free group included (1) patients free from metastases after the removal of primary melanoma, (2) patients with complete remission after pharmacological treatment, and (3) patients free from metastasis after lymph node block dissection.

### 2.2. Enzyme-Linked Immunosorbent Assay

Plasma osteopontin protein levels were determined using the ELISA technique using commercially available Human Osteopontin (OPN) Quantikine ELISA Kit (R & D Systems, Inc., Minneapolis, MN, USA, Catalog#: DOST00). Assay range of the kit is 0.3–20 ng/mL; sensitivity is 0.024 ng/mL. The assay was carried out in accordance with the manufacturer’s protocol. Briefly, the plasma samples were diluted twenty-fivefold with calibrator diluent. First, 100 μL of assay diluent was added to each well, then 50 µL of osteopontin standard or sample were added and incubated for 2 h at room temperature (RT). After washing the plate four times, 200 μL of human OPN conjugate was added to each well and incubated for 2 h at RT. Then, the washing steps were repeated four times and 200 μL of substrate solution was added to each well. The samples were incubated for 30 min at RT in dark, and 50 μL of stop solution was added to each well. Finally, the optical density of each well was determined at 540 nm using Epoch™ Microplate Spectrophotometer (BioTek Instruments, Winooski, VT, USA).

### 2.3. Statistical Analysis

The normality of the data was determined using the Shapiro–Wilk test. χ^2^-test or Fisher exact tests were applied to analyze the categorical variables. In cases of a normal distribution, independent *t*-tests (two-tailed) were used to compare the continuous variables, otherwise Mann–Whitney test was applied.

Univariate and multivariate logistic regression analyses were performed to create a diagnostic prediction model. Odds ratio (OR) was calculated with corresponding 95% upper and lower confidence intervals. Melanoma patients were randomly allocated into training and validation sets in a 2:1 ratio. In the training and validation sets, the optimal cut-off values for discriminating the presence or absence of metastasis was calculated using receiver operating characteristic (ROC) curve analysis in cases of variables and combined variables. The optimal cut-off was defined using the maximum Youden index. The area under the curve (AUC) derived from ROC curve was computed with 95% confidence intervals (95% CI). In general, an AUC of 0.5 suggests no discrimination, 0.7 to 0.8 is considered acceptable, 0.8 to 0.9 is considered excellent, and more than 0.9 is considered outstanding. Furthermore, sensitivity, specificity, positive predictive value (PPV), negative predictive value (NPV), and diagnostic accuracy were calculated with 95% confidence intervals.

The significance level was *p* < 0.05 in all cases. Statistically significant differences are denoted by *, **, and *** for *p* < 0.05, *p* < 0.01, and *p* < 0.001. Statistical analyses were performed with IBM SPSS Statistics for Windows, version 25.0 (IBM Corp., Armonk, NY, USA) and R software (version 3.6.4; R Foundation for Statistical Computing, Vienna, Austria). Statistical analysis was supervised by a biostatistician, E.A. Janka.

## 3. Results

### 3.1. Patients and Melanoma Characteristics

**Figure 1:**
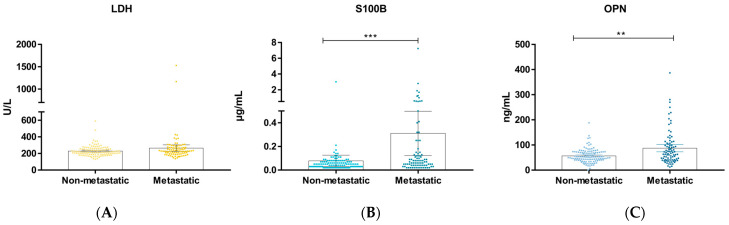
Comparison of biomarkers in the non-metastatic and metastatic melanoma groups. (**A**) Serum LDH levels in the non-metastatic and metastatic melanoma groups. (**B**) Serum S100B levels in the non-metastatic and metastatic melanoma groups. (**C**) Plasma OPN levels in the non-metastatic and metastatic melanoma groups. The dots show the values, while the gray box shows the mean with a 95% confidence interval. LDH—lactate dehydrogenase; OPN—osteopontin; ** *p* < 0.001; *** *p* < 0.001.

Altogether, 206 melanoma patients, 120 patients with non-metastatic, and 86 with metastatic melanoma were included in our study. Between the two groups there was no significant difference in the ratio of sex (*p* = 0.317). The average age of the non-metastatic patients was 61.00 ± 12.28 years, while in the metastatic group it was 64.14 ± 11.68 years (*p* = 0.092). The primary tumor characteristics showed that the most frequent subtype was nodular melanoma in both groups. Furthermore, the more invasive (Clark level V) and thicker than 4 mm ulcerated primary tumor (pT4b) occurred significantly more frequently in the metastatic group. The presence of the BRAF mutation did not differ significantly between the groups (*p* = 0.375) (Table 1). Serum S100B and osteopontin levels were significantly higher in the metastatic group (*p* < 0.001 and *p* = 0.002, respectively); however, serum LDH levels showed no significant difference (*p* = 0.107) between the non-metastatic and metastatic melanoma patient groups (Table 1, Figure 1).

### 3.2. Univariate and Multivariate Analysis

Logistic regression models were used to assess the factors influencing the diagnosis of metastasis in melanoma patients. First, in the univariate logistic regressions, the primary tumor location, American Joint Committee on Cancer (AJCC) 8th edition pT category, and serum S100B, LDH, and plasma OPN levels showed a significant effect on the probability of metastasis (Table 2). The significant factors were implemented in the multivariate logistic regression model.

In the multivariate logistic regression model, the location of the primary tumor, more specifically the location of the primary tumor on the lower extremities, and the AJCC 8th edition pT2b-pT3a, pT3b-pT4a, and pT4b categories, as well as the S100B and osteopontin levels, were associated with the diagnosis of metastasis (Table 2).

### 3.3. Training Set and Validation Set

The 206 melanoma patients were randomly divided into a training and a validation set in a 2:1 ratio in order to check the diagnostic model. The patient characteristics of the training (N = 138) and validation (N = 68) set are presented in Table 1. Neither in the training set nor in the validation set did age, sex, histological subtype, or BRAF mutational status show significant differences between the non-metastatic and metastatic melanoma groups. The primary tumor location, Clark level, and pT stage were significantly different between the metastatic and non-metastatic melanoma patients in the training set but not in the validation set. In both the training and validation sets, a significant difference was detected between the metastatic and non-metastatic groups for serum S100B and plasma OPN levels, but there was no significant difference for serum LDH levels (Table 1).

### 3.4. Diagnostic Effect of Biomarkers and the Combinations

**Figure 2:**
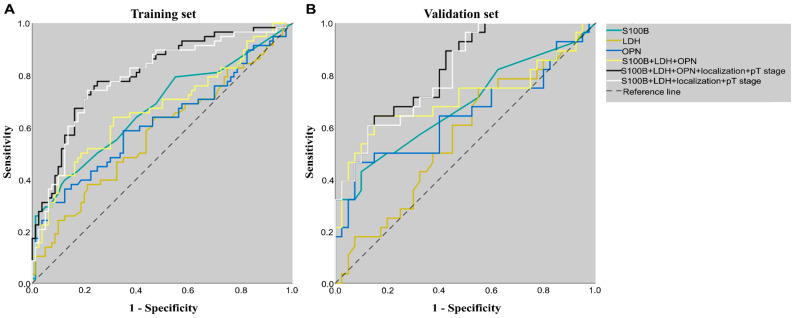
ROC curves of biomarkers and different combination. (**A**) ROC curves of single, combined biomarkers, and combined models in the training set. (**B**) ROC curves of single, combined biomarkers, and combined models in the validation set. ROC—receiver operating curve; LDH—lactate dehydrogenase; OPN—osteopontin; pT stage—American Joint Committee on Cancer 8th edition T category.

An ROC analysis was used to discover the optimal cut-off value for biomarkers and to determine the AUC. The optimal cut-off value for the serum S100B was 0.085 µg/L and the AUROC was 0.671 [0.598; 0.752] in the training set and 0.682 [0.547; 0.817] in the validation set (Table 3, Figure 2). For the serum LDH, the optimal cut-off was 220.5 U/L, and the AUC was only 0.575 [0.477; 0.674] in the training set and 0.563 [0.424; 0.703] in the validation set. In the case of osteopontin, the optimal cut-off value of 80.09 ng/mL was determined using the Youden index, and the AUROC was 0.616 [0.518; 0.715] in the training set and 0.643 [0.500; 0.786] in the validation set (Table 3, Figure 2). The combination of the blood biomarkers resulted in similar AUC values (Table S1).

The combination of the three blood biomarkers, the primary tumor localization, and the AJCC 8th edition pT category resulted in the highest AUC values (0.803 [0.729; 0.878]) in both the training set and the validation set (0.822 [0.726; 0.919]). Omitting the OPN level from this combined model still resulted in strong AUC values for both the training and validation sets, with 0.791 [0.713; 0.869] and 0.812 [0.712; 0.911], respectively (Table 3, Figure 2).

## 4. Discussion

The 5-year survival rate of metastatic melanoma is 31–34% with targeted combined BRAF–MEK inhibitor therapy, 42–43% with anti-PD1 inhibitor therapy, and 46–57% with combined anti-CTLA-4 and anti-PD-1 therapy [14,15,16].

Today, there is a growing need for blood biomarkers to monitor high risk patients or those with metastatic melanoma in order to improve survival. Of course, from stage IIB of melanoma, initial and regular follow-up imaging examinations are warranted and preferably computed using tomography scans [17].

The LDH is a non-secreted cytoplasmic enzyme. Therefore, an increased serum concentration is associated with tumor necrosis in patients with advanced stage melanoma. The elevated LDH levels have also been shown to be associated with an unfavorable prognosis [8]. In a study involving 121 patients, the specificity of LDH for confirming the presence of regional or distant metastases (stage III-IV. patients) was 92.2% and its sensitivity was 41.9% [18]. In the study by Bánfalvi et al., LDH exhibited a sensitivity of 41.6%, a specificity of 80%, a positive predictive value of 95.9%, and a negative predictive value of 10.9% in patients with stage III–IV melanoma [19]. Since LDH levels can be elevated in diseases other than melanoma, false-positive cases are more common. Furthermore, it has also been demonstrated to be less sensitive in the early stages compared to patients with an advanced disease course [20]. In our study, serum LDH levels exhibited a significant effect (*p* = 0.019) on the odds ratio of metastasis in univariate logistic regression analysis; however, this effect did not hold in the multivariate logistic regression model. The discriminative power, as indicated by the AUC, for serum LDH was only 0.575.

S100B is a calcium-binding protein and it belongs to the S100 protein family, playing a crucial role in various intracellular processes [21], such as regulating protein phosphorylation and degradation, dynamic changes in cytoskeleton components, activating enzymes and transcription factors, and influencing cell proliferation, differentiation, and inflammation. Additionally, it is involved in the regulation of intracellular calcium homeostasis.

In melanoma patients, elevated serum S100B levels result from the cell death and protein degradation caused by apoptosis or necrosis. These levels correlate with the stage of melanoma: lower levels are typically measured in stages I and II, while serum levels increase in more advanced cases [21]. Furthermore, a significant correlation has been observed between the Breslow score and the serum S100B levels in patients with stage III melanoma. Patients with tumor infiltration into deeper tissue layers had higher S100B concentrations [20,22], with the highest values detected in stage IV. Thus, serum S100B may not serve as a reliable marker for the early diagnosis of melanoma. However, it has proven to be valuable in monitoring patients and detecting progression in asymptomatic patients. It is worth noting that while S100B offers higher specificity for melanoma compared to LDH, elevated S100B levels have been measured in various other conditions, including cardiovascular diseases, liver cirrhosis, migraine, breast carcinoma, vitiligo, SARS-CoV-2 infection, brain, liver, and kidney injury [20,23,24,25]. Additionally, serum S100B levels tend to be elevated in central nervous system-related pathological conditions, neurodegenerative diseases, certain psychiatric disorders, and in cases of brain tumors and brain metastases, including melanoma brain metastases [21]. The early detection of relapse and progression holds significant importance for the prognosis, as delayed detection often leads to poorer chances of survival. Measuring serum S100B levels can be useful for early detection of relapse. In fact, the first study comparing S100B and different imaging techniques for predicting a melanoma relapse was conducted in 2006 involving a total of 192 melanoma patients [10]. All these patients shared a common feature of experiencing relapse after a tumor-free period. Among them, 127 patients had concurrent S100B measurements in parallel with imaging. Notably, 37% of these patients had elevated S100B levels at the time of relapse. In stage III patients, 32% had elevated serum levels, and during progression to stage IV this value was increased to 48%. Importantly, for 5.5% of the patients, an elevated S100B level was the initial sign of progression. In contrast, the imaging only detected melanoma recurrence in 26.8% of cases. These findings strongly suggest that S100B serves as a specific and sensitive marker for detecting melanoma progression [10]. In 2011, a study was published comparing the significance of S100B and PET-CT in patients with a metastatic melanoma [26]. According to these observations, the measurement of serum S100B in the follow-up of patients with melanoma is a valuable tool for detecting disease progression in asymptomatic individuals. Its diagnostic value is further enhanced when these measurements are followed by PET-CT scans [26]. Another study demonstrated that S100B has a sensitivity of 54.1%, a specificity of 93.3%, a positive predictive value of 98.9%, and a negative predictive value of 15.3% for stage III–IV [19]. Elevated S100B levels have been associated with significantly worse survival in melanoma patients [20,27]. Nevertheless, the questions remain open about the validity of measuring serum S100B levels for detecting melanoma relapse [21], and there is still no consensus on the necessity for peripheral blood sampling in the follow-up of melanoma patients [10,28,29,30].

In a 2021 meta-analysis, the prognostic (predicting survival) and diagnostic (predicting relapse) significance of serum S100B and serum LDH in patients with melanoma was compared [31]. Regarding the diagnostic impact, elevated S100B levels emerged as a more reliable marker for predicting relapse in melanoma patients at various stages compared to elevated LDH levels. In terms of the prognostic impact, no significant difference was observed between elevated S100B and elevated LDH levels, both of which were associated with a higher risk of death. These markers can be considered independent prognostic factors [31].

In our study, serum S100B levels exhibited a significant effect on the probability of metastasis in both univariate (*p* < 0.001) and multivariate (*p* = 0.020) logistic regression analysis. Moreover, the discriminative power of serum S100B in identifying metastatic disease was stronger than that of serum LDH, although the AUC did not reach 0.7, even for serum S100B. Notably, we identified that the ROC-optimized cut-off for serum S100B was lower than the manufacturer’s cut-off. This is important because the cut-off value influences the sensitivity and specificity established for the diagnostic test. In contrast, the ROC-optimized cut-off for serum LDH matched the upper limit of normal (ULN), as determined by the local laboratory.

Our results confirm the necessity of exploring additional blood biomarkers for the more reliable diagnosis of metastases in melanoma patients.

OPN is a glycophosphoprotein consisting of 314 amino acids. It contains several highly conserved binding sites that enable binding to the cell surface and other molecules [32,33]. Through these interactions, it plays a crucial role in tumorigenesis and progression, as well as the regulation of various signaling pathways including MAPK, NF-kB pathway, and ERK signaling. It exhibits an increased expression in osteoblasts, osteoclasts, skeletal muscle cells, endothelial cells, and certain carcinoma cells [32,33]. The exact mechanism of its action in tumor development and progression is still unclear. However, it plays a crucial role in various processes, including cell adhesion and migration, immune response regulation, extracellular matrix (ECM) interaction, and it serves as a determinant of tumor cell motility, angiogenesis, tumor growth, inhibition of apoptosis, invasion, and metastasis [32,33]. Studies demonstrated that osteopontin expression, both at the mRNA and protein levels, is elevated in thick melanomas and associated with ulceration [11,34]. Furthermore, significantly increased osteopontin (mRNA) expression has been observed in cell lines derived from melanoma metastases in comparison to those cell lines developed from primary melanomas. Additionally, a recent study found significantly elevated osteopontin gene expression in melanoma cell lines carrying the BRAFV600E mutation [12]. In another study, plasma concentrations of OPN were significantly higher in patients with metastatic melanoma compared to non-metastatic patients [13].

We obtained similar results in our patient population, with significantly higher levels of OPN (*p* = 0.002) in the metastatic group compared to the non-metastatic group. Additionally, plasma OPN levels showed a significant impact on the odds of metastasis in both univariate (*p* < 0.001) and multivariate (*p* < 0.001) logistic regression analysis. The discriminative power of plasma OPN in identifying metastatic disease was not stronger than that of serum S100B, and the AUC values were similar for serum S100B and serum OPN.

Our study revealed that the primary tumor’s location in the lower extremities and the AJCC 8th edition pT2b-pT3a, pT3b-pT4a, and T4b categories were independent determinants of an increased probability of metastasis. Notably, factors such as age, sex, histological subtype, and BRAF mutation status did not exhibit a significant impact on the odds of metastasis. To enhance the diagnostic effect of identifying metastatic disease, we examined the combination of the three blood biomarkers with primary tumor localization and the AJCC 8th edition pT category. Our findings indicated that the inclusion of the primary tumor localization and pT category substantially improved the discriminative power of all blood biomarkers in identifying metastatic disease (AUC~0.8). The AUC was the strongest in the model combining serum S100B, LDH, OPN, tumor localization, and T category. However, while serum S100B measurement is routinely available in most hospitals, plasma OPN measurement is not. The AUC was similarly strong when OPN level was omitted from the combined model.

## 5. Conclusions

In summary, our results suggest that a combined evaluation of the primary tumor location, the AJCC 8th edition pT category, and the serum S100B and LDH levels can serve as a reliable marker of metastasis prediction. Plasma osteopontin can be classified as a melanoma biomarker, and by combining clinicopathological prognostic variables, the diagnostic effect of blood biomarkers in the detection of metastatic melanoma can be improved.

However, it is important to acknowledge several limitations of this study. First, this was a single-center retrospective analysis and the sample size was not large, limiting the analysis of different melanoma subgroups. A larger number of samples is needed, for example, to further analyze the diagnostic effect of histological subtypes. Although data on *BRAF* mutational status were available in most of the primary or metastatic tumors, not all cases had this information. Additionally, in many cases, the 95% confidence intervals in the logistic regression models had wide ranges, underscoring the need for further analysis with a larger patient group.

## Figures and Tables

**Table 1:** Characteristics of melanoma population.

	Patients N = 206			Training Set N = 138			Validation Set N = 68		
Variables	Non-Metastatic N = 120	Metastatic N = 86	*p*-Value	Non-Metastatic N = 80	Metastatic N = 58	*p*-Value	Non-Metastatic N = 40	Metastatic N = 28	*p*-Value
Melanoma patients									
Age (years) (mean ± SD)	61.00 ± 12.28	64.14 ± 11.68	0.092	60.86 ± 12.15	64.67 ± 12.56	0.076	61.15 ± 12.73	63.96 ± 9.53	0.754
Sex, N (%)			0.317			0.493			0.444
Male	67 (55.8)	54 (62.8)		45 (56.3)	36 (62.1)		22 (55.0)	18 (64.3)	
Female	53 (44.2)	32 (37.2)		35 (43.7)	22 (37.9)		18 (45.0)	10 (35.7)	
Primary melanoma									
Localization, N (%)			0.108			**0.007**			0.395
Head and neck	16 (13.3)	11 (12.8)		14 (17.5)	6 (10.3)		2 (5.0)	5 (17.9)	
Upper extremities	33 (27.5)	28 (32.6)		19 (23.7)	20 (34.5)		14 (35.0)	8 (28.6)	
Lower extremities	21 (17.5)	24 (27.9)		13 (16.3)	20 (34.5)		8 (20.0)	4 (14.3)	
Trunk	50 (41.7)	23 (26.7)		34 (42.5)	12 (20.7)		16 (40.0)	11 (39.2)	
Histological subtype, N (%)			**0.019**			0.098			0.130
SSM	44 (22.5)	16 (30.2)		28 (35.0)	11 (19.0)		16 (40.0)	5 (17.9)	
NM	49 (40.8)	44 (51.2)		33 (41.3)	27 (46.6)		16 (40.0)	17 (60.7)	
MM	27 (36.7)	26 (18.6)		19 (23.7)	20 (34.4)		8 (20.0)	6 (21.4)	
Clark level, N (%)			**0.012**			**0.024**			0.344
II	10 (8.3)	2 (2.3)		7 (8.8)	2 (3.4)		3 (7.5)	0 (0.0)	
III	50 (41.7)	27 (31.4)		31 (38.7)	18 (31.0)		19 (47.5)	9 (32.1)	
IV	42 (35.0)	23 (26.8)		31 (38.7)	15 (25.9)		11 (27.5)	8 (28.6)	
V	10 (8.3)	18 (20.9)		4 (5.0)	11 (19.0)		6 (15.0)	7 (25.0)	
Unknown	8 (6.7)	16 (18.6)		7 (8.8)	12 (20.7)		1 (2.5)	4 (14.3)	
AJCC 8th edition pT Category, N (%)			**<0.001**			**0.001**			0.061
pT1a-T2a	43 (35.8)	8 (9.3)		30 (37.5)	6 (10.3)		13 (32.5)	2 (7.1)	
pT2b-T3a	18 (15.0)	14 (16.3)		11 (13.7)	10 (17.2)		7 (17.5)	4 (14.3)	
pT3b-T4a	25 (20.8)	18 (20.9)		18 (22.5)	12 (20.7)		7 (17.5)	6 (21.4)	
pT4b	34 (28.4)	46 (53.5)		21 (26.3)	30 (51.7)		13 (32.5)	16 (57.1)	
*BRAF* mutation									
*BRAF*			0.375			0.141			0.578
Wild type	33 (27.5)	53 (61.6)		21 (26.2)	39 (67.2)		12 (30.0)	14 (50.0)	
V600E/K	27 (22.5)	32 (37.2)		19 (23.8)	19 (32.8)		8 (20.0)	13 (46.4)	
Unknown	60 (50.0)	1 (1.1)		40 (50.0)	0		20 (50.0)	1 (3.6)	
Biomarkers									
LDH (U/L) (mean ± SD)	229.10 ± 61.55	264.63 ± 180.55	0.107	228.45 ± 55.28	279.52 ± 215.51	0.132	230.70 ± 73.25	233.79 ± 55.78	0.380
S100B (µg/mL) (mean ± SD)	0.08 ± 0.27	0.31 ± 0.87	**<0.001**	0.09 ± 0.33	0.34 ± 1.01	**<0.001**	0.05 ± 0.03	0.25 ± 0.48	**0.010**
OPN (ng/mL) (mean ± SD)	56.63 ± 27.09	87.33 ± 67.64	**0.002**	55.65 ± 28.30	86.89 ± 73.67	**0.019**	58.59 ± 24.70	88.26 ± 54.27	**0.046**

Significant results are in bold. N—number; SD—standard deviation; SSM—superficial spreading melanoma; NM –nodular melanoma; MM—unclassified malignant melanoma or histological subtype other than SSM or NM; AJCC—American Joint Committee on Cancer; LDH—lactate dehydrogenase; OPN—osteopontin.

**Table 2:** Univariate and multivariate logistic regression analysis for metastatic melanoma.

		Univariate Logistic Regression Model		Multivariate Logistic Regression Model	
Variables	Categories	OR [95% CI]	*p*-Value	OR [95% CI]	*p*-Value
Age	≥60 years/<60 years	1.22 [0.69; 2.17]	0.487	-	
Sex	male/female	1.34 [0.76; 2.35]	0.318	-	
Localization of primary tumor	head and neck/trunk	1.50 [0.60; 3.72]	0.388	2.14 [0.73; 6.26]	0.166
	upper extremities/trunk	1.85 [0.91; 3.73]	0.089	2.30 [0.99; 5.34]	0.052
	lower extremities/trunk	**2.48 [1.16; 5.35]**	**0.020**	**2.80 [1.14; 6.90]**	**0.025**
Histological subtype	SSM/MM	0.38 [0.17; 1.10]	0.065	-	
	NM/MM	0.93 [0.48; 1.83]	0.839		
Clark level	III/II	2.70 [0.55; 13.22]	0.220	-	
	IV/II	2.74 [0.55; 13.58]	0.218		
	V/II	9.00 [0.86; 49.47]	0.110		
AJCC 8th edition pT category	pT2b-T3a/pT1a-T2a	**4.18 [1.50; 11.69]**	**0.006**	**4.18 [1.25; 14.02]**	**0.020**
	pT3b-T4a/pT1a-T2a	**3.87 [1.47; 10.19]**	**0.006**	**4.09 [1.32; 12.70]**	**0.015**
	pT4b/pT1a-T2a	**7.27 [3.03; 17.45]**	**<0.001**	**9.86 [3.51; 27.67]**	**<0.001**
*BRAF*	V600E/K/wild type	0.74 [0.38; 1.45]	0.375	-	
S100B		**3.29 [1.81; 5.98]**	**<0.001**	**2.30 [1.14; 4.63]**	**0.020**
LDH		**2.55 [1.17; 5.59]**	**0.019**	1.91 [0.72; 5.06]	0.193
OPN		**4.95 [2.45; 10.02]**	**<0.001**	**5.13 [2.21; 11.91]**	**<0.001**

Significant results are in bold. OR—odds ratio; 95% CI—95% confidence intervals, SSM—superficial spreading melanoma; NM—nodular melanoma; MM—unclassified malignant melanoma or histological subtype other than SSM or NM; AJCC—American Joint Committee on Cancer; LDH—lactate dehydrogenase; OPN—osteopontin.

**Table 3:** Diagnostic effect of biomarkers and the combinations.

		Training Set						Validation Set					
Variables	Cut-Off	AUROC [95% CI] *p*-Value	Sensitivity [95% CI]	Specificity [95% CI]	PPV [95% CI]	NPV [95% CI]	Diagnostic Accuracy [95% CI]	AUROC [95% CI] *p*-Value	Sensitivity [95% CI]	Specificity [95% CI]	PPV [95% CI]	NPV [95% CI]	Diagnostic Accuracy [95% CI]
S100B	0.085	0.671 [0.598; 0.752], ***p* = 0.001**	0.43 [0.30; 0.57]	0.83 [0.72; 0.90]	0.64 [0.51; 0.76]	0.67 [0.61; 0.72]	0.66 [0.57; 0.74]	0.682 [0.547; 0.817] ***p* = 0.011**	0.43 [0.25; 0.63	0.90 [0.76; 0.97]	0.75 [0.52; 0.89]	0.69 [0.62; 0.76]	0.71 [0.58; 0.81]
LDH	220.5	0.575 [0.477; 0.674] *p* = 0.131	0.64 [0.50; 0.76]	0.53 [0.41; 0.64]	0.67 [0.57; 0.75]	0.43 [0.42; 0.57]	0.57 [0.49; 0.66]	0.563 [0.424; 0.703] *p* = 0.376	0.54 [0.34; 0.73]	0.55 [0.39; 0.71]	0.46 [0.34; 0.58]	0.63 [0.51; 0.73]	0.54 [0.42; 0.67]
OPN	80.09	0.616 [0.518; 0.715] ***p* = 0.020**	0.36 [0.24; 0.50]	0.88 [0.78; 0.94]	0.65 [0.61; 0.70]	0.68 [0.52; 0.81]	0.66 [0.57; 0.74]	0.643 [0.500; 0.786] ***p* = 0.046**	0.46 [0.28; 0.66]	0.90 [0.76; 0.97]	0.77 [0.54; 0.90]	0.71 [0.63; 0.78]	0.72 [0.60; 0.82]
S100B + localization + pT stage	0.435	0.788 [0.709; 0.867] ***p* < 0.001**	0.76 [0.63; 0.86]	0.76 [0.65; 0.85]	0.70 [0.60; 0.78]	0.81 [0.73; 0.87]	0.76 [0.68; 0.83]	0.802 [0.699; 0.904] ***p* < 0.001**	0.61 [0.41; 0.79]	0.80 [0.64; 0.91]	0.68 [0.52; 0.81]	0.74 [0.64; 0.83]	0.72 [0.60; 0.82]
LDH + localization + pT stage	0.458	0.785 [0.706; 0.864] ***p* < 0.001**	0.72 [0.59; 0.83]	0.79 [0.68; 0.87]	0.71 [0.61; 0.80]	0.80 [0.72; 0.86]	0.76 [0.68; 0.83]	0.744 [0.624; 0.864] ***p* = 0.001**	0.71 [0.51; 0.87]	0.75 [0.59; 0.87]	0.67 [0.53; 0.78]	0.79 [0.67; 0.87]	0.74 [0.61; 0.84]
OPN + localization + pT stage	0.460	0.791 [0.714; 0.868] ***p* < 0.001**	0.67 [0.54; 0.79]	0.80 [0.70; 0.88]	0.71 [0.60; 0.80]	0.77 [0.70; 0.83]	0.75 [0.67; 0.82]	0.798 [0.694; 0.902] ***p* < 0.001**	0.64 [0.44; 0.81]	0.80 [0.64; 0.91]	0.69 [0.53; 0.82]	0.76 [0.66; 0.84]	0.74 [0.61; 0.84]
S100B + LDH + OPN + localization + pT stage	0.413	0.803 [0.729; 0.878] ***p* < 0.001**	0.78 [0.65; 0.88]	0.75 [0.64; 0.84]	0.69 [0.60; 0.77]	0.82 [0.74; 0.88]	0.76 [0.68; 0.83]	0.822 [0.726; 0.919] ***p* < 0.001**	0.68 [0.48; 0.84]	0.78 [0.62; 0.89]	0.68 [0.53; 0.80]	0.78 [0.66; 0.86]	0.74 [0.61; 0.84]
S100B + LDH + localization + pT stage	0.459	0.791 [0.713; 0.869] ***p* < 0.001**	0.74 [0.61; 0.85]	0.79 [0.68; 0.87]	0.72 [0.62; 0.80]	0.81 [0.73; 0.87]	0.77 [0.69; 0.84]	0.812 [0.712; 0.911] ***p* < 0.001**	0.61 [0.41; 0.79]	0.85 [0.70; 0.94]	0.74 [0.56; 0.86]	0.76 [0.66; 0.83]	0.75 [0.63; 0.85]

Significant results are in bold. AUROC—area under the receiver operating characteristics, PPV—positive predictive value, NPV—negative predictive value, 95% CI—95% confidence intervals, LDH—lactate dehydrogenase, OPN—osteopontin, pT stage—American Joint Committee on Cancer 8th edition T category.

## Data Availability

Data are contained within the article.

## Supplementary Materials

The following supporting information can be downloaded at: https://www.mdpi.com/article/10.3390/jcm13010174/s1, Table S1: Diagnostic effect of biomarkers and the combinations.
