# Supplementary material for: Combining Biomarkers for the Diagnosis of Metastatic Melanoma
Source: J Clin Med. 2023 Dec 28;13(1):174. doi: 10.3390/jcm13010174 (PMC10779676; doi:10.3390/jcm13010174)
Supplement: Supplementary file 1 [file jcm-13-00174-s001.zip › jcm-2738054-supplementary.pdf]

## Supplementary materials

**Supplementary Table S1.** Diagnostic effect of biomarkers and the combinations

| Variables | Cut-off | Training set | | | | | | Validation set | | | | | |
|---|---|---|---|---|---|---|---|---|---|---|---|---|---|
| | | AUROC [95% CI] p-value | Sensitivity [95% CI] | Specificity [95% CI] | PPV [95% CI] | NPV [95% CI] | Diagnostic accuracy [95% CI] | AUROC [95% CI] p-value | Sensitivity [95% CI] | Specificity [95% CI] | PPV [95% CI] | NPV [95% CI] | Diagnostic accuracy [95% CI] |
| S100B+LDH | 0.443 | 0.650 [0.555; 0.746] **p=0.003** | 0.38 [0.26; 0.52] | 0.90 [0.81; 0.96] | 0.73 [0.57; 0.85] | 0.67 [0.62; 0.71] | 0.68 [0.60; 0.76] | 0.687 [0.547; 0.828] **p=0.009** | 0.50 [0.31; 0.69] | 0.85 [0.70; 0.94] | 0.70 [0.51; 0.84] | 0.71 [0.62; 0.78] | 0.71 [0.58; 0.81] |
| S100B+OPN | 0.465 | 0.658 [0.560; 0.755] **p=0.002** | 0.41 [0.29; 0.55] | 0.90 [0.81; 0.96] | 0.75 [0.59; 0.86] | 0.68 [0.63; 0.73] | 0.70 [0.61; 0.77] | 0.709 [0.575; 0.843] **p=0.004** | 0.54 [0.34; 0.73] | 0.83 [0.67; 0.93] | 0.68 [0.50; 0.82] | 0.72 [0.63; 0.80] | 0.71 [0.58; 0.81] |
| LDH+OPN | 0.437 | 0.643 [0.549; 0.738] **p=0.004** | 0.45 [0.32; 0.59] | 0.80 [0.70; 0.88] | 0.62 [0.49; 0.73] | 0.67 [0.61; 0.72] | 0.65 [0.57; 0.73] | 0.643 [0.500; 0.785] **p=0.046** | 0.46 [0.28; 0.66] | 0.83 [0.67; 0.93] | 0.65 [0.46; 0.80] | 0.69 [0.60; 0.76] | 0.68 [0.55; 0.79] |
| S100B+LDH +OPN | 0.391 | 0.669 [0.575; 0.763] **p=0.001** | 0.64 [0.50; 0.76] | 0.69 [0.57; 0.79] | 0.58 [0.50; 0.68] | 0.72 [0.64; 0.79] | 0.67 [0.58; 0.75] | 0.712 [0.573; 0.850] **p=0.003** | 0.64 [0.44; 0.81] | 0.78 [0.62; 0.89] | 0.67 [0.51; 0.79] | 0.76 [0.65; 0.84] | 0.72 [0.60; 0.82] |

Significant results are in bold. AUROC – area under the receiver operating characteristics. PPV- positive predictive value. NPV – negative predictive value. 95% CI – 95% confidence intervals, LDH – lactate dehydrogenase; OPN – osteopontin
